# Synthesis and Characterization of Organotin Containing Copolymers: Reactivity Ratio Studies

**DOI:** 10.3390/molecules15031784

**Published:** 2010-03-12

**Authors:** Salem S. Al-Deyab, Ali Mohsen Al-Hazmi, Mohamed H. El-Newehy

**Affiliations:** Petrochemical Research Chair, Chemistry Department, College of Science, King Saud University, Riyadh 11451, P.O. Box 2455, Saudi Arabia; E-Mails: ssdeyab@ksu.edu.sa (S.S.A.); petrochem@ksu.edu.sa (A.M.A.)

**Keywords:** organotin monomers, citraconate, maleate, styrene, butyl acrylate, reactivity ratio

## Abstract

Organotin monomers containing dibutyltin groups – dibutyltin citraconate (DBTC) as a new monomer and dibutyltin maleate (DBTM) – were synthesized. Free radical copolymerizations of the organotin monomers with styrene (ST) and butyl acrylate (BA) were performed. The overall conversion was kept low (≤15% wt/wt) for all studied samples and the copolymers composition was determined from tin analysis using the Gillman and Rosenberg method. The reactivity ratios were calculated from the copolymer composition using the Fineman-Ross (FR) method. The synthesized monomers were characterized by elemental analysis, ^1^H-, ^13^C-NMR and FTIR spectroscopy.

## 1. Introduction

Copolymerization is one of the most important means to improve the performance of polymers. Copolymers are extensively used in industrial processes, because their physical properties, such as elasticity, permeability, glass transition temperature (*T_g_*) and solvent diffusion kinetics can be varied within wide limits [1,2]. Knowledge of a copolymer’s composition is an important factor in the evaluation of its utility [3,4]. Controlling the polymer property parameters, such as copolymer composition, copolymer sequence distribution and molecular weight averages, is of particular importance in copolymerization processes. This is because copolymer density and viscosity, which are two of the most important property measures used by polymer manufacturers, depend on these parameters [5]. Reactivity ratios are among the most important parameters for the composition equation of copolymers, which can offer information such as the relative reactivity of monomer pairs and help estimate the copolymer composition [3]. To calculate the polymerization rate or polymer productivity and copolymer composition, monomer reactivity ratios must be known. The method which is used most often nowadays for estimating monomer reactivity ratios is to perform a low conversion copolymerization at various initial monomers feed compositions. Subsequently, the copolymer composition is determined for each reaction [5]. Reactivity ratio values may be evaluated by various procedures: linear procedures, nonlinear procedures, and other copolymer composition equations [6,7,8,9].

Organotin derivatives of a compound containing bioactive alkyltin groups have considerable interest as biocides [10]. Organotin compounds have important applications in several areas and hence they are made industrially on a large scale [11]. The organotin moiety is attached to the monomers and copolymers *via* O-Sn and/or N-Sn bonds [10,11,12,13,14,15]. Acrylic copolymers with pendant organotin moieties find widespread applications as antifouling agents, [10,16] wood preservatives, [10] fungicides, pesticides, mosquito larvacides, [10,17] heat and light stabilizers in the manufacture of poly(vinyl chloride). [10] and biological activities against various species [10,18].

The present article investigates the synthesis, and structural characterization of copolymers of dibutyltin citraconate (DBTC), and dibutyltin maleate (DBTM), with styrene (ST) and butyl acrylate (BA) as well as the reactivity ratios in the copolymerization. For this purpose, reactivity ratios for the classical copolymerization model were determined using the linearization methods of Finemann–Ross (FR method) [19].

## 2. Results and Discussion

### 2.1. Synthesis of Organotin Monomers

**Scheme 1:**
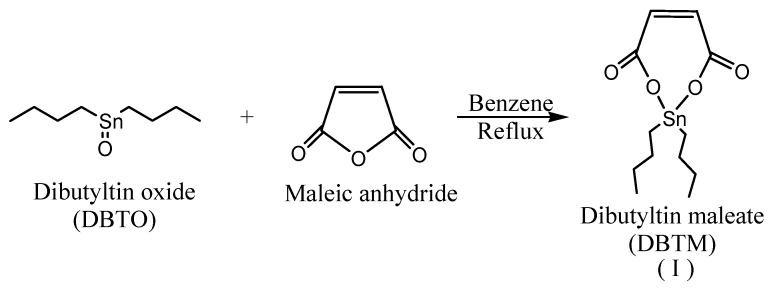
Synthesis of dibutyl maleate (DTBM, **I**).

**Scheme 2:**
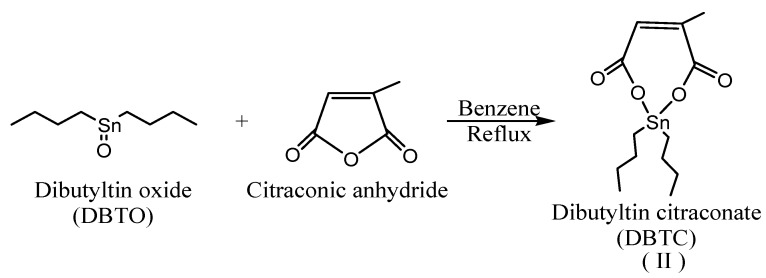
Synthesis of dibutyltin citraconate (DBTC, II)

Organotin monomers, (I) and (II), were prepared by the reaction of dibutyltin oxide (DBTO) with maleic anhydride or citraconic anhydride in equimolecular ratio as shown in Scheme 1 and Scheme 2. 

The structures were elucidated by elemental analysis, FTIR, ^1^H and ^13^C-NMR spectroscopy. In addition, the purity of the prepared monomers was checked by Thin Layer Chromatography (TLC) using ethyl acetate/cyclohexane (2:1). Generally, elemental microanalyses, as shown in Table 1, are in a good agreement with the calculated values.

#### 2.1.1. Dibutyltin Maleate (DBTM, **I**)

The FT-IR spectrum of dibutyltin maleate (DBTM, **I**) showed characteristic peaks at: 2,854, 2,868, 2,926, and 2,958 cm^-1^ assigned to C-H stretching (-CH_2_CH_2_CH_2_CH_3_, and -CH=CH-), 1,615 cm^-1^ assigned to C=O stretching and 1,582 cm^-1^ assigned to C=C stretching. The ^1^H-NMR spectrum (CDCl_3_) showed peaks at δ 0.86 (triplet, -CH_2_CH_2_CH_2_C*H_3_*), δ 1.30–1.36 (multiplet, -CH_2_C*H_2_*CH_2_CH_3_), δ 1.65 (multiplet, -C*H_2_*CH_2_CH_2_CH_3_), δ 1.74 (triplet, -CH_2_CH_2_C*H_2_*CH_3_), δ 6.22 (singlet, -C*H*=C*H*-). The ^13^C-NMR spectrum (CDCl_3_) showed peaks at δ 13.60, 25.79, 26.54, 26.73 (-*C*H_2_*C*H_2_*C*H_2_*C*H_3_), δ 129.61 (-*C*H=*C*H-), δ 175.01 (-*C*=O).

#### 2.1.2. Dibutyltin Citraconate (DBTC, **II**)

The FT-IR spectrum of dibutyltin citroconate (DBTC, **II**) showed peaks at: 2,857, 2,868, 2,927, and 2,956 cm^-1^ assigned to C-H stretching (-CH_2_CH_2_CH_2_CH_3_, & -CH=CH-), 1,610 and 1,667 cm^-1^ assigned to C=O stretching, 1,561 cm^-1^ assigned to C=C stretching. The ^1^H-NMR spectrum showed peaks at δ 0.80 (triplet, -CH_2_CH_2_CH_2_C*H_3_*), δ 1.26 (multiplet, -CH_2_C*H_2_*CH_2_CH_3_), δ 1.59 (multiplet, -C*H_2_*CH_2_CH_2_CH_3_), δ 1.61 (triplet, -CH_2_CH_2_C*H_2_*CH_3_), δ 1.96 (singlet, -CH=C-C*H_3_*), δ 5.77 (singlet, -C*H*=C-CH_3_). The ^13^C-NMR spectrum showed peaks at δ 13.59, 25.46, 26.62, 26.71 (-*C*H_2_*C*H_2_*C*H_2_*C*H_3_), δ 20.81 (-CH=C-*C*H_3_), δ 120.91 (-*C*H=C-CH_3_), δ 145.22 (-CH=*C*-CH_3_), δ 174.80 (-CH=CCH_3_-*C*=O), δ 177.92 (-*C*O-CH=C-CH_3_).

### 2.2. Copolymerization Method

**Scheme 3:**
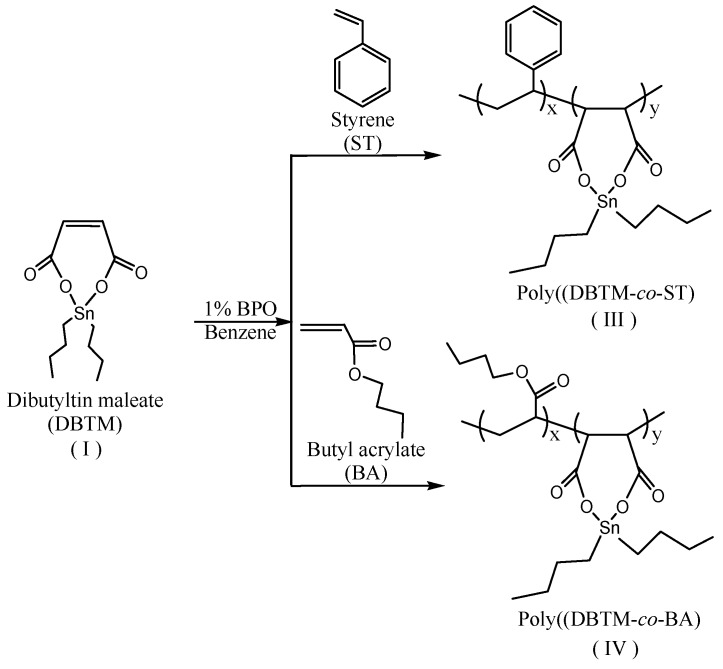
Copolymerization of DBTM with ST and BA.

**Scheme 4:**
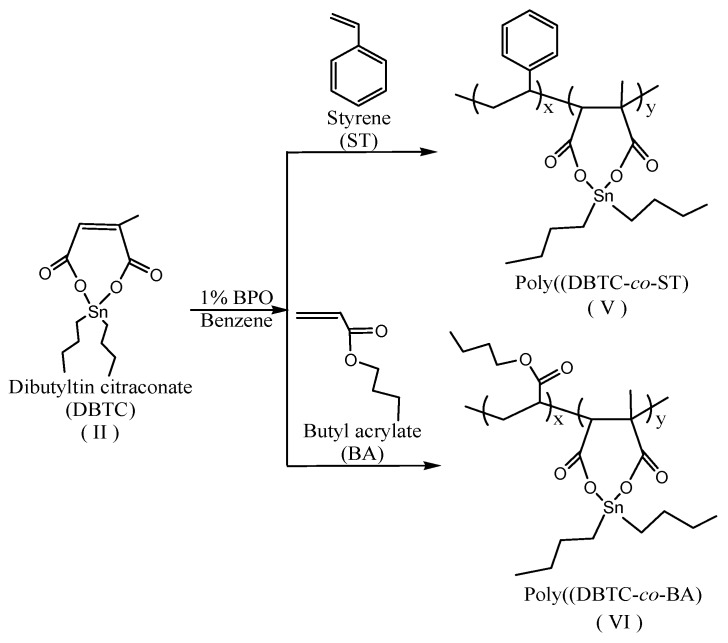
Copolymerization of DBTC with ST and BA.

Copolymerization of DBTM or DBTC with styrene (ST) and butyl acrylate (BA) was done in solution using benzoyl peroxide as initiator *via* the free radical technique (Scheme 3 and Scheme 4).

Copolymerization was done at 70 ºC in benzene with a total concentration of 2 mol/L at different time intervals. The formed copolymer was precipitated in an excess amount (20 fold), of the corresponding solvent, and was purified by washing with excess precipitation solvent or by reprecipitation from benzene, chloroform, or acetone, depending on the type of copolymer. All samples were dried in an oven under vacuum at 40–60 ºC. Different copolymers with different ratios were prepared and the percentage of tin was determined in each sample [20] (Table 2).

### 2.3. Overall Conversion and Structural Characterization

The aim of the copolymerization was to study the copolymerization behavior of the monomers DBTM and DBTC, with ST and BA. The overall conversion of monomers to poly(DBTM-*co*-ST) (**III**), poly(DBTM-*co*-BA) (**IV**), poly(DBTC-*co*-ST) (**V**) and poly(DBTC-*co*-BA) (**VI**), after 2 h, was found to be 4.09, 34.01%, 4.52 and 24.93%, respectively. After 8 h, the overall conversion of monomers to (**III**), (**IV**), (**V**) and (**VI**) was found to be 13.89, 36.8%, 16.55 and 38.40%, respectively. 

So the copolymerization of DBTM or DBTC with BA showed the highest overall conversion, compared to ST. Based on the percentage of tin (Table 3), the copolymer composition showed that the content of DBTM in (**III**) is higher than in (**IV**) and the copolymer composition showed that the content of DBTC in (**V**) is lower than in (**VI**) after 4 h [21].

The structural characterizations of (**III**), (**IV**), (**V**) and (**VI**) were done by FTIR and ^1^H-NMR spectroscopy. The FTIR spectrum of (**III**), (**IV**), (**V**) and (**VI**) with overall conversion after 8 hrs of 13.89, 36.80, 16.55 and 38.40%, respectively, was characterized by the disappearance of C=C stretching bands at 1,582 and 1,610–1,640 cm^-1^ of DBTM or DBTC with ST or BA, respectively which confirm the formation of the copolymer.

Generally, the FTIR spectrum showed peaks at 1,452, 1,492, 1,583, and 1,601 cm^-1^ assigned to C=C stretching of the aromatic ring of ST [8]. The FTIR spectrum also showed peaks at 3,024, 3,058, and 3,080 cm^-1^ assigned to C-H stretching of the aromatic ring, and peaks at 2,848, 2872, 2931, 2,914, and 2957cm^-1^ assigned to aliphatic C-H stretching. On the other hand, the FTIR showed characteristic peaks at 1,605 and 1,736 cm^-1^ assigned to C=O stretching of DBTM and BA, respectively.

The ^1^H-NMR spectrum of (**III**) and (**IV**) was characterized by the disappearance of peaks at δ 5.49–6.23 ppm (-C*H*=C*H*- and C*H_2_*=C*H*-) or (-C*H*=CCH_3_- and C*H_2_*=C*H*-) of DBTM or DBTC with ST or BA, respectively, which confirm the formation of the copolymer.

The ^1^H-NMR spectrum of (**III**) was characterized by the appearance of peaks at δ 0.91–2.17 ppm (-C*H_2_*C*H_2_*C*H_2_*C*H_3_*, -C*H_2_*-CPh-, and -C*H*-C*H*-), and at δ 6.58–7.25 ppm (*H_arom_*). The ^1^H-NMR spectrum of (**IV**) was characterized by the appearance of peaks at δ 0.92–1.59 ppm (-C*H_2_*C*H_2_*C*H_2_*C*H_3_*, -COOCH_2_C*H_2_*C*H_2_*C*H_3_*, -C*H*-CHCOO-) and at δ 4.03 ppm (-C*H*-C*H*-, -CH-C*H*COO-).

The ^1^H-NMR spectrum of (**V**) was characterized by the appearance of peaks at δ 0.90–1.83 ppm (-C*H_2_*C*H_2_*C*H_2_*C*H_3_*, -C*H_2_*-C-COO-, and -CH-CHC*H_3_*-), and at δ 6.57–7.25 ppm (*H_arom_*). The ^1^H-NMR spectrum of (**VI**) was characterized by the appearance of peaks at δ 0.89–1.57 ppm (-C*H_2_*C*H_2_*C*H_2_*C*H_3_*, -COOCH_2_C*H_2_*C*H_2_*C*H_3_*, -C*H*-CHCOO-, -CH-CHC*H_3_*-) and at δ 4.01 ppm (-C*H*-C*H*CH_3_-, -CH-C*H*COO-).

### 2.4. Reactivity Ratio Determination

#### 2.4.1. Poly(DBTM-co-ST) (**III**)

Poly(DBTM-*co*-ST) (**III**) was prepared using different ratios of DBTM and ST with BPO as initiator and the polymerization was stopped at an overall conversion ≤15 wt/wt%. The copolymer was precipitated in excess methanol. The percentage of tin was calculated according to the Gilman and Rosenberg method [20], and subsequently the copolymer composition (f) was determined as shown in (Table 4). The monomers reactivity ratios and the content of the reaction mixture and the copolymer was calculated according to the FR method [22,23,24] (Table 5). The FR parameters for DBTM and ST (Table 6) were calculated by plotting the relation between F(f-1)/f and F_2_/f. From the values of the experimental reactivity ratio, r_1_ (*k_11_/k_12_*) is smaller than r_2_ (*k_22_/k_21_*), it is evident that monomer DBTM (r_1_ = 0.099) is less reactive towards the addition of its units compared to the addition of ST units. On the other hand, the route of ST (r_2_ = 9.9065) is more reactive towards the addition of its units compared to the addition of DBTM units. As r_1_r_2_ < 1, so the copolymer tends to random distribution of its monomer units [15].

#### 2.4.2. Poly(DBTM-co-BA) (**IV**)

Poly(DBTM-*co*-BA) (**IV**) was prepared using different ratios of DBTM and BA with BPO as initiator and the polymerization was stopped at an overall conversion ≤15 wt/wt%. The copolymer was precipitated in excess methanol and was purified by reprecipitation in chloroform. The percentage of tin was calculated according to the Gilman and Rosenberg method [20], and subsequently the copolymer composition (f) was determined as shown in Table 7. The monomers reactivity ratios and the content of the reaction mixture and the copolymer was calculated according to FR method [12,13,14] (Table 8). The FR parameters for DBTM and BA (Table 6) were calculated by plotting the relation between F(f-1)/f and F_2_/f. From the values of the experimental reactivity ratio, r_1_ (*k_11_/k_12_*) is smaller than r_2_ (*k_22_/k_21_*), it is evident that monomer DBTM (r_1_ = 0.0248) is less reactive towards the addition of its units compared to the addition of BA units. On the other hand, the route of BA (r_2_ = 24.431) is more reactive towards the addition of its units compared to the addition of DBTM units. As r_1_ < 1 and r_2_ > 1, so the copolymer will contain blocks of BA with low random units of DBTM due to the high reactivity of BA with its high reactivity ratio compared to DBTM [25]. Moreover, as r_1_r_2_ < 1, so the copolymer tends to random distribution of its monomer units.

#### 2.4.3. Poly(DBTC-co-ST) (**V**)

Poly(DBTC-*co*-ST) (**V**) was prepared using different ratios of DBTC and ST using BPO as initiator and the copolymerization was stopped at overall conversion ≤15 wt/wt%. The copolymer was precipitated in excess methanol. The percentage of tin was calculated according to the Gilman and Rosenberg method [20], and subsequently the copolymer composition (f) was determined (Table 9). The monomers reactivity ratios and the content of the reaction mixture and the copolymer was calculated according to FR method [22,23,24] (Table 10). The FR parameters for DBTC and ST (Table 6) were calculated by plotting the relation between F(f-1)/f and F_2_/f.

From the values of the experimental reactivity ratio, r_1_ (*k_11_/k_12_*) is smaller than r_2_ (*k_22_/k_21_*), it is evident that the monomer ST prefers the addition of its units compared to the addition of DBTC units. As r_1_ < 1 and r_2_ > 1, so the copolymer will contain blocks of ST with low random units of DBTC due to the high reactivity of ST with its high reactivity ratio compared to DBTC [25]. Finally, when r_1_ < 1, the copolymerization is preferred and when r_2_ > 1, ST will tend to homopolymerizations.

#### 2.4.4. Poly(DBTC-co-BA) (VI)

Poly(DBTC-*co*-BA) (VI) was prepared using different ratios of DBTC and BA using BPO as initiator and the polymerization was stopped at overall conversion ≤15 wt/wt%. The copolymer was precipitated in excess methanol and was purified by reprecipitation from acetone. The percentage of Tin was calculated according to Gilman and Rosenberg method [20], and subsequently the copolymer composition (f) was determined as shown in (Table 11). The monomers reactivity ratios and the content of the reaction mixture and the copolymer was calculated according to FR method [22,23,24] (Table 12). The FR parameters for DBTC and BA (Table 6) were calculated by plotting the relation between F(f-1)/f and F_2_/f.

From the values of the experimental reactivity ratio, r_1_ (*k_11_/k_12_*) is smaller than r_2_ (*k_22_/k_21_*), it is evident that monomer DBTC (r_1_ = 0.2727) is less reactive towards the addition of its units compared to the addition of BA units. On the other hand, the route of BA (r_2_ = 33.611) is more reactive towards the addition of its units compared to the addition of DBTC units. As r_1_ < 1 and r_2_ > 1, so the copolymer will contain blocks of BA with low random units of DBTC due to the high reactivity of BA with its high reactivity ratio compared to DBTC [25]. Moreover, when r_1_ < 1, the copolymerization is preferred and when r_2_ > 1, BA will tend to homopolymerization.

## 3. Experimental

### 3.1. Materials

Dibutyltin (IV) oxide was purchased from Sigma-Aldrich. Citraconic anhydride and styrene (ST) were purchased from Fluka. Maleic anhydride 99% and benzoyl peroxide (BPO) were purchased from BDH. Butyl acrylate and 2, 2’-azobisisobutyronitrile (AIBN) were purchased from Riedel-de-Haen. All solvents were purchased from BDH and were used as received.

### 3.2. Characterization

^1^H- and ^13^C-NMR Spectra were recorded on a Jeol (400 MHz) instrument. FTIR Spectra were recorded on a Perkin Elmer 883. Elemental analyses were performed at Perkin Elmer Series II CHN/O Analyzer 2400. Thin-layer chromatography (TLC) was performed using the ascending technique with silica gel 60F 254 precoated aluminum sheets.

### 3.3. Synthesis of Organotin Monomers

#### 3.3.1. Synthesis of Dibutyltin Maleate (DBTM, **I**)

In a 500 mL round bottom flask, maleic anhydride (4.90 g, 50.0 mmol) was added to dibutyltin oxide (12.44 g, 50.0 mmol) in dry benzene (170 mL). The mixture was heated under gentle reflux for 9 h. The formed precipitate was removed by filtration, and the solvent was totally evaporated on a rotavapor to give an oily residue. The oily residue was dissolved in diethyl ether (100 mL) under gentle heating with stirring. The solution was filtered and the filtrate was concentrated to one third its volume on a rotavapor. The solution was cooled to room temperature and then was kept in freezer (-10 ºC) for 72 h to give a white precipitate. The formed precipitate was filtered, recrystallized from diethyl ether [26] and was dried under vacuum at 40 ºC for 24 h to give 10.20 g, 58.8% yield and m.p. 127–129 ºC. The product **I** was characterized by elemental analysis (Table 1), FTIR and ^1^H- and ^13^C NMR spectroscopy.

#### 3.3.2. Synthesis of Dibutyltin Citraconate (DBTC, **II**)

The dibutyltin citraconate (DBTC, **II**) was prepared as described earlier for DBTM (**I**) using the following quantities and conditions: dibutyltin oxide (12.44 g, 50.0 mmol), citraconic anhydride (5.60 g, 50.0 mmol) in dry benzene (170 mL) for 10 hrs. The product **II** was recrystallized from *n*-hexane and was dried under vacuum at 40 ºC for 24 h to give 9.10 g, 50.4% yield and m.p. 107–110 ºC. The product **II** was characterized by elemental analysis (Table 1), FTIR and ^1^H- and ^13^C-NMR spectroscopy.

### 3.4. General Procedure for Copolymerization

Copolymerizations were carried out in a three neck round bottomed flask. Copolymerization was done in solution by dissolving benzoyl peroxide (BPO) (1% mol) in 2 mL of the corresponding solvent, and then the calculated molar quantities of the monomers were added. The reaction mixture was bubbled with nitrogen to expel oxygen. Copolymerization was done at 70 ºC for the desired period of time. The formed copolymer was precipitated in excess amount (20 fold), of the corresponding solvent. All samples were dried in oven under vacuum at 40–60 ºC. For reactivity ratio determination, the copolymerization was stopped at overall conversion below 15% wt/wt [25] from the total weight of both monomers by changing the time of polymerization.

### 3.5. Overall Conversion

The overall conversion in copolymerization [25,27,28] of monomers DBTM and DBTC with ST and BA was studied by taking a fixed number of moles (20 mmol), and composition of 20% mol of monomer DBTM or DBTC and 80% mol of ST and BA, in benzene with a total concentration of 2 mol/L at different time intervals. The overall conversion by weight (wt/wt%) was determined using Equation (1):
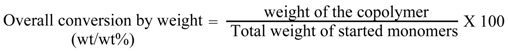
(1)

### 3.6. Reactivity Ratios Determination

For reactivity ratio determination, copolymerizations were performed with different initial feed ratios while maintaining the monomer conversion below 15%. The Fineman–Ross (FR) method was employed. The initiator concentration was kept at 1% relative to the total monomers concentration in benzene. Monomer reactivity ratios can be calculated from the experimental results depending on the copolymer composition. Copolymer composition can be expressed as following;

f_1_ = m_1_/m_2_     and    f_2_ = m_2_/m_1_

Where m_1_ & m_2_ are the mole fractions of DBTM or DBTC and vinyl monomer in the copolymer, respectively, and f_1_ & f_2_ are its molar ratios in the copolymer.

Moreover, the feed composition of the reaction mixture is known in advance, so feed composition was used in the calculations of the reactivity ratios and can be expressed as follows:

F_1_ = M_1_/M_2_     and     F_2_ = M_2_/M_1_

where M_1_ & M_2_ are the mole fractions of DBTM or DBTC and vinyl monomer in the reaction mixture, respectively, and F_1_ & F_2_ are its Molar ratios in the feed composition.

In this research, the calculations were based on the tin content in the copolymer composition [29]. The Fineman–Ross (FR) [30] method is based on the use of copolymer composition and the content of the polymerization mixture. Based on the calculations of the copolymer composition and feed composition and according to equation (2):
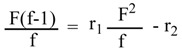
(2)

A plot of (F^2^/f) on X-axis *vs* {F/f(f-1)} on Y-axis gave a straight line, the intercept is r_2_ and the slope is r_1_.

## 4. Conclusions

The organotin monomers dibutyltin citraconate (DBTC) as a new monomer, and dibutyltin maleate (DBTM), were synthesized. The organotin monomers were copolymerized with styrene (ST) and butyl acrylate (BA) using a free radical technique. The overall conversion was kept low (≤15% wt/wt) for all studied samples and the copolymers composition was determined from tin analysis. From the values of the experimental reactivity ratio, r_1_ (*k_11_/k_12_*) is smaller than r_2_ (*k_22_/k_21_*), it is evident that poly(DBTM-*co*-ST) (**III**), poly(DBTM-*co*-BA) (**IV**) tend to random distribution of its monomer units as r_1_r_2_ < 1. For poly(DBTC-*co*-ST) (**V**), r_1_ < 1 and r_2_ > 1, so the copolymer will contain blocks of ST with low random units of DBTC due to the high reactivity of ST with its high reactivity ratio compared to DBTC. For poly(DBTC-*co*-BA) (**VI**), r_1_ < 1 and r_2_ > 1, so the copolymer will contain blocks of BA with low random units of DBTC due to the high reactivity of BA with its high reactivity ratio compared to DBTC.

## Figures and Tables

**Table 1:** Elemental microanalysis of synthesized monomers **I** and **II**.

Monomer	*Calc.*			Found		
	%C	%H	%Sn	%C	%H	%Sn*
**I**	41.54	5.81	34.21	41.85	6.01	33.11
**II**	43.25	6.14	32.88	42.85	6.56	33.11

*Sn was estimated using the Gilman and Rosenberg method [20].

**Table 2:** Tin percentage of copolymers (III-VI) with different ratios.

Copolymer Ratio	%Sn			
	III^a^	IV^b^	V^c^	VI^d^
80/20	15.00	7.14	15.39	7.99
60/40	11.47	4.18	10.68	4.50
50/50	9.00	2.88	7.66	2.93
40/60	6.02	2.33	5.03	2.06
30/70	2.67	0.73	2.01	0.72

^a^ DBTM/ST; ^b^ DBTM/BA; ^c^ DBTC/ST; ^d^ DBTC/BA

**Table 3:** Copolymers composition after 4 h.

Copolymer code	%Sn	Moles of				F ^a^
		DBTM	DBTC	ST	BA	
III	3.74	0.0314	-	0.9011	-	0.0367
IV	1.19	0.0100	-	-	0.7531	0.0133
V	1.19	-	0.0426	0.8450	-	0.0225
VI	3.74	-	0.0165	-	0.7338	0.0504

^a^ Copolymer Composition.

**Table 4:** The parameters of poly(DBTM-*co*-ST) (III) composition.

Copolymer Ratio	%Sn	M_1_^a^	F^b^	m_1_^c^	f^d^	Conversion (wt/wt%)^e^
20/80	2.67	0.2	0.2500	0.0247	0.0253	4.45
40/60	6.02	0.4	0.6667	0.0601	0.0639	3.79
50/50	9.00	0.5	1.0000	0.0964	0.1067	4.48
60/40	11.47	0.6	1.5000	0.1310	0.1507	4.60
70/30	15.00	0.7	2.3330	0.1891	0.2333	3.81

^a^ Mole fraction of DBTM in reaction mixture; ^b^ Molar ratio of DBTM to ST in reaction mixture; ^c^ Mole fraction of DBTM in copolymer; ^d^ Molar ratio of DBTM to ST in copolymer; ^e^ Overall conversion.

**Table 5:** The monomer reactivity ratios and the FR parameters of poly(DBTM-*co*-ST) (**III**).

Copolymer Ratio	Monomer Ratio F = M_1_/M_2_	M-Unit Ratio in Copolymer	Parameters of FR Eq.	
			F^2^/f	F/f(f-1)
20/80	0.25	0.0253	2.4714	-9.6355
40/60	0.667	0.0639	6.9520	-9.7609
50/50	1.0	0.1067	9.3687	-8.3687
60/40	1.5	0.1507	14.9250	-8.4503
70/30	2.333	0.1137	23.3340	-7.6686

**Table 6:** Experimental reactivity ratios according to the FR method.

Copolymer	r_1_(*k_11_/k_12_*) ^a^	r_2_(*k_21_/k_22_*) ^b^	r_1_r_2_
**III**	0.0990	9.0650	0.9807
**IV**	0.0248	24.4310	0.7058
**V**	0.3258	14.8030	4.8228
**VI**	0.2727	33.6110	9.1657

^a^ Reactivity Ratio of DBTM or DBTC; ^b^ Reactivity Ratio of vinyl monomers (ST and BA).

**Table 7:** The parameters of poly(DBTM-*co*-BA) (IV) composition.

Copolymer Ratio	%Sn	M_1_^a^	F ^b^	m_1_^c^	F ^d^	Conversion (wt/wt%)^f^
20/80	0.73	0.2	0.250	0.0080	0.0080	7.23
40/60	2.33	0.4	0.6667	0.0263	0.0270	6.49
50/50	2.88	0.5	1.000	0.0327	0.0338	8.01
60/40	4.18	0.6	1.500	0.0487	0.0512	7.19
70/30	7.14	0.7	2.333	0.0886	0.0972	1.84

^a^ Mole fraction of DBTM in reaction mixture; ^b^ Molar ratio of DBTM to BA in reaction mixture; ^c^ Mole fraction of DBTM in copolymer; ^d^ Molar ratio of DBTM to BA in copolymer; ^f^ Overall conversion.

**Table 8:** The monomers reactivity ratios and the FR parameters of poly(DBTM-*co*-BA) (IV).

Copolymer Ratio	Monomer Ratio F = M_1_/M_2_	M-Unit Ratio in Copolymer	Parameters of FR Eq.	
			F^2^/f	F/f(f-1)
20/80	0.250	0.0080	7.7895	-30.0908
40/60	0.667	0.0027	16.4899	-24.0554
50/50	1.000	0.0338	29.5598	-28.5598
60/40	1.500	0.0512	43.9158	-27.7772
70/30	2.333	0.0972	67.7638	-26.7532

**Table 9:** The parameters of poly(DBTC-*co*-ST) (V) composition.

Copolymer Ratio	%Sn	M_1_^a^	F ^b^	m_1_^c^	F ^d^	Conversion (wt/wt%)^e^
20/80	2.01	0.2	0.250	0.0184	0.0188	3.15
40/60	5.03	0.4	0.667	0.0494	0.0520	4.09
50/50	7.66	0.5	1.000	0.0803	0.0873	4.03
60/40	10.68	0.6	1.500	0.1214	0.1382	3.76
70/30	15.39	0.7	2.333	0.2016	0.2525	2.15

^a^ Mole fraction of DBTC in reaction mixture; ^b^ Molar ratio of DBTC to ST in reaction mixture; ^c^ Mole fraction of DBTC in copolymer; ^d^ Molar ratio of DBTC to ST in copolymer; ^e^ Overall conversion.

**Table 10:** The monomers reactivity ratios and the FR parameters of poly(DBTC-*co*-ST) (V).

Copolymer Ratio	Monomer Ratio F = M_1_/M_2_	M-Unit Ratio in Copolymer	Parameters of FR Eq.	
			F^2^/f	F/f(f-1)
20/80	0.250	0.0188	3.3318	-13.0773
40/60	0.667	0.0520	8.5572	-12.1624
50/50	1.000	0.0873	11.454	-10.4544
60/40	1.500	0.1382	16.276	-9.3509
70/30	2.333	0.2525	21.552	-6.9049

**Table 11:** The parameters of poly(DBTC-co-BA) (VI) composition.

Copolymer Ratio	%Sn	M_1_^a^	F^b^	m_1_^c^	f^d^	Conversion (wt/wt%)^e^
20/80	0.72	0.2	0.250	0.0079	0.0079	13.38
40/60	2.06	0.4	0.667	0.0231	0.0237	4.49
50/50	2.93	0.5	1.000	0.0343	0.0355	6.82
60/40	4.50	0.6	1.500	0.0532	0.0562	3.00
70/30	7.99	0.7	2.333	0.1021	0.1137	1.56

^a^ Mole fraction of DBTC in reaction mixture; ^b^ Molar ratio of DBTC to BA in reaction mixture; ^c^ Mole fraction of DBTC in copolymer; ^d^ Molar ratio of DBTC to BA in copolymer; ^f^ Overall conversion.

**Table 12:** The monomer reactivity ratios and the FR parameters of poly(DBTC-co-BA) (VI).

Copolymer Ratio	Monomer Ratio F = M1/M2	M-Unit Ratio in Copolymer	Parameters of FR Eq.	
			F2/f	F/f(f-1)
20/80	0.250	0.0079	7.8379	-31.1018
40/60	0.667	0.0237	18.795	-27.5115
50/50	1.000	0.0355	28.132	-27.1319
60/40	1.500	0.0562	40.051	-25.2009
70/30	2.333	0.1137	47.888	-18.1932
